# Peripheral Innate Immune Activation Correlates With Disease Severity in *GRN* Haploinsufficiency

**DOI:** 10.3389/fneur.2019.01004

**Published:** 2019-09-18

**Authors:** Peter A. Ljubenkov, Zachary Miller, Paige Mumford, Jane Zhang, Isabel Elaine Allen, Laura Mitic, Adam Staffaroni, Hilary Heuer, Julio C. Rojas, Yann Cobigo, Anna Karydas, Rodney Pearlman, Bruce Miller, Joel H. Kramer, Michael S. McGrath, Howard J. Rosen, Adam L. Boxer

**Affiliations:** ^1^Department of Neurology, Memory and Aging Center, University of California, San Francisco, San Francisco, CA, United States; ^2^Department of Laboratory Medicine, University of California, San Francisco, San Francisco, CA, United States; ^3^Department of Epidemiology and Biostatistics, University of California, San Francisco, San Francisco, CA, United States; ^4^The Bluefield Project to Cure Frontotemporal Dementia, San Francisco, CA, United States; ^5^Department of Medicine, University of California, San Francisco, San Francisco, CA, United States

**Keywords:** progranulin (GRN), frontotemperal lobar degeneration, monocyte, innate immune system, peripheral immune activation

## Abstract

**Objective:** To investigate associations between peripheral innate immune activation and frontotemporal lobar degeneration (FTLD) in progranulin gene (*GRN*) haploinsufficiency.

**Methods:** In this cross-sectional study, ELISA was used to measure six markers of innate immunity (sCD163, CCL18, LBP, sCD14, IL-18, and CRP) in plasma from 30 *GRN* mutation carriers (17 asymptomatic, 13 symptomatic) and 29 controls. Voxel based morphometry was used to model associations between marker levels and brain atrophy in mutation carriers relative to controls. Linear regression was used to model relationships between plasma marker levels with mean frontal white matter integrity [fractional anisotropy (FA)] and the FTLD modified Clinical Dementia Rating Scale sum of boxes score (FTLD-CDR SB).

**Results:** Plasma sCD163 was higher in symptomatic *GRN* carriers [mean 321 ng/ml (SD 125)] compared to controls [mean 248 ng/ml (SD 58); *p* < 0.05]. Plasma CCL18 was higher in symptomatic *GRN* carriers [mean 56.9 pg/ml (SD 19)] compared to controls [mean 40.5 pg/ml (SD 14); *p* < 0.05]. Elevation of plasma LBP was associated with white matter atrophy in the right frontal pole and left inferior frontal gyrus (*p* FWE corrected <0.05) in all mutation carriers relative to controls. Plasma LBP levels inversely correlated with bilateral frontal white matter FA (R^2^ = 0.59, *p* = 0.009) in mutation carriers. Elevation in plasma was positively correlated with CDR-FTLD SB (b = 2.27 CDR units/μg LBP/ml plasma, R^2^ = 0.76, *p* = 0.003) in symptomatic carriers.

**Conclusion:** FTLD-*GRN* is associated with elevations in peripheral biomarkers of macrophage-mediated innate immunity, including sCD163 and CCL18. Clinical disease severity and white matter integrity are correlated with blood LBP, suggesting a role for peripheral immune activation in FTLD-*GRN*.

## Introduction

Haploinsufficiency of the progranulin gene (*GRN*) is a major cause of familial frontotemporal lobar degeneration (FTLD), giving rise to a variety of fatal and untreatable frontotemporal dementia (FTD-*GRN*) syndromes (1). Progranulin has a broad role in vertebrates, including regulation of lysosomal function, angiogenesis, blood monocytes, and brain microglia (2, 3). In patients with FTD-*GRN*, there is an increased rate of autoimmunity (4), suggesting that peripheral immune dysregulation is a feature of this disease. In *Grn*^−/−^ mice, peripheral myeloid cells and microglia release excessive pro-inflammatory cytokines in response to bacterial lipopolysaccharide (LPS), and both sets of mononuclear cells exhibit heightened neurotoxicity (3). Moreover, *Grn*^−/−^ mice display poor reconstitution of the blood brain barrier after injury (5). If humans recapitulate animal models of familial FTLD, patients with *GRN* deficiency are likely to have hyperactive monocytes, with less restricted access to the central nervous system (CNS) and greater capacity for neuronal injury. Plasma biomarkers of innate immune activation may therefore provide evidence for a potentially treatable monocyte-driven mechanism of pathogenesis in GRN deficiency and serve as measures of drug response in future clinical therapeutic trials targeting myeloid cells. Additionally, given the documented relationship between peripheral inflammation and white matter integrity outside of FTD cohorts (6), abnormal peripheral monocyte activation may serve to explain the unique burden of white matter disease that distinguishes FTD-*GRN* from other familial FTD syndromes (7).

**Figure 1:**
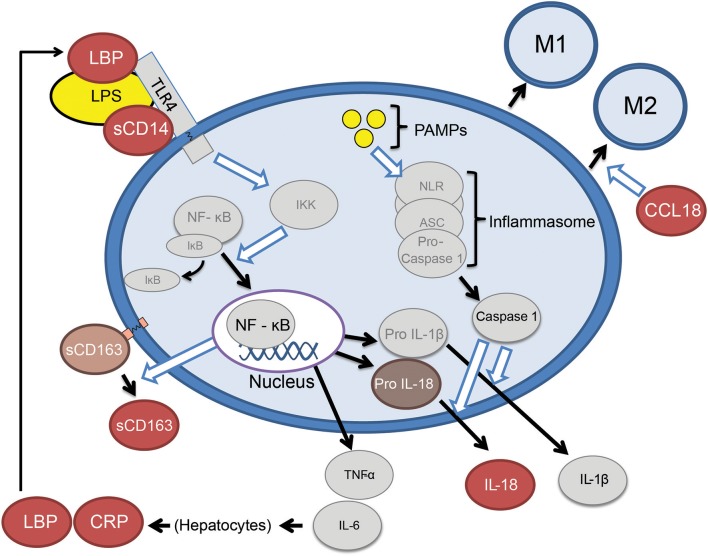
Abbreviated summary of monocyte activation pathways. Proteins depicted as red ovals were selected as candidate markers of monocyte activation. Bacterial lipopolysaccharide (LPS) requires the presence of LBP (lipopolysaccharide binding protein) and sCD14 (soluble cluster of differentiation 14), to TLR4 (toll-like receptor 4) and the NF-κB (nuclear factor kappa-light-chain-enhancer of activated B cells) (8). Activating monocytes cleave surface CD163 (Cluster of Differentiation 163) in to a soluble form (sCD163) (9). Monocytes may also be activated by pathogen-associated molecular patterns (PAMPS) which lead to release IL-18 via the inflammasome pathway (10). Classical monocytes (CD14^+^, CD16^−^) eventually transition to intermediate (CD14^+^CD16^+^) and eventually non-classical monocytes (CD14^low^, CD16^+^) (11), the latter of which are a source of CCL18 (chemokine C-C motif ligand 18) (12).

Six specific candidate plasma biomarkers of innate immune activation were selected for investigation in patients with *GRN* mutations (Figure 1). Lipopolysaccharide binding protein (LBP) and soluble Cluster of Differentiation 14 (sCD14) (8) were selected as candidate biomarkers due to their roles as important cofactors in toll-like receptor 4 (TLR4) activation via bacterial endotoxin (a trigger of excessive myeloid activation in *Grn*^−/−^ mice) (3). In contrast to LPS and sCD14, plasma interleukin 18 (IL18), a marker of the inflammasome pathway (10), was selected as a marker of monocyte activation via a TLR4 independent pathway. Soluble cluster of differentiation 163 (sCD163), a protein cleaved from recently activated monocytes, was selected as an established general marker of ongoing monocyte activation and turnover (9). Additionally, chemokine (C-C motif) ligand 18 (CCL18), a marker of non-classical (potentially anti-inflammatory) monocytes (12), was selected as indicator of chronic phenotypic transition away from classical monocyte forms (a shift common to many chronic inflammatory states). Finally, C-reactive protein (CRP), an acute phase reactant, was also assessed as a non-specific biomarker of immune activation. Our approach provides early evidence that peripheral innate immune activation is a feature of *GRN* haploinsufficiency and a potential mediator of FTD-*GRN* pathogenesis.

## Methods

### Patient Selection

Patient demographics are summarized in Table 1. 30 individuals with known *GRN* mutations (17 asymptomatic and 13 symptomatic) and 29 age-matched controls were recruited through the University of California San Francisco Memory and Aging Center between 2009 and 2016. Our controls were not family members of mutation carriers. Patients were classified as “symptomatic” if they met criteria for mild cognitive impairment (13) or experienced loss of functional independence from a neurodegenerative syndrome. Within the symptomatic *GRN* mutation carrier cohort, seven individuals met consensus criteria for bvFTD (14), one met criteria for mild cognitive impairment (13) (with a progressive dysexecutive/behavioral syndrome), three met consensus criteria for a PPA (15), one had an amnestic syndrome resembling Alzheimer's disease (with a negative amyloid PET scan), one presented with idiopathic parkinsonism, and one individual suffered from a multifactorial dementia syndrome with behavioral, memory, language, and visuospatial impairments. Among the three participants who met criteria for PPA, one met criteria for the non-fluent variant of PPA (nfvPPA), and two presented with mixed PPA syndromes which could not be further subcategorized. This array of heterogeneous clinical syndromes was consistent with the known clinical heterogeneity of symptomatic GRN haploinsufficiency (1). Participants included in this study did not have any documented history of immunosuppression, autoimmune disease, hypothyroidism, acute infection, chronic infection, or positive biomarkers consistent with Alzheimer's pathology (including cerebrospinal fluid Amyloid Beta 42 or Amyloid beta PET with an FDA approved ligand) on retrospective review of their medical history. Only one *GRN* mutation carrier was excluded from our analyses, due to a positive amyloid PET scan. One control participant was also excluded from our analyses, due to the presence of autoimmune disease requiring immunosuppression. This study was approved by the UCSF Institutional Review Board (IRB), and all participants gave informed consent.

**Table 1:** Patient demographics, plasma biomarkers, and radiographic measures.

	**Controls**	***GRN*** **mutation carriers**	
		**Asymptomatic**	**Symptomatic**
Total (*N*)	29	17	13
Volumetric MRI (*N*)	22	14	11
DTI imaging (*N*)	17	12	10
Female/male	16/13	8/9	7/6
	**Mean (SD)**	**Mean (SD)**	**Mean (SD)**
Age	57.4 (13)	54.4 (11)	62(7)
**Plasma markers**			
Plasma sCD163 (ng/ml)	248 (58)	273 (91)	321 (125)^†^
Plasma CCL18 (pg/ml)	40.5 (14)	54.2 (26)	56.9 (19)^†^
Plasma sCD14 (μg/ml)	1.60 (2)	1.45 (0.3)	1.68 (3)
Plasma LBP (μg/ml)	9.62 (5)	8.92 (2)	9.57 (2)
Plasma CRP (μg/ml)	2.07 (3)	1.80 (2)	1.82 (2)
Plasma IL18 (pg/ml)	26.3(12)	25.4 (9)	30.2 (17)
**Clinical measures**			
CDR-FTLD SB	0.06 (0.2)	0.64 (0.8)^†^	8.7 (7)^†^^‡^
FAQ total	0 (0)	0.6 (1.9)	14.1 (12)^†^^‡^
CGIS	1 (0)	1.2 (0.4)	4.0 (1)^†^^‡^
MMSE total	29.4 (1)	28.9 (1)	19.3 (10)^†^^‡^
CVLT immediate recall	8.4 (0.8)	7.8 (1)	5.1 (3)^†^^‡^
CVLT delayed recall	8.4 (0.9)	7.7 (1)	5.0 (3)^†^^‡^
CVLT D^1^	3.3 (0.3)	3.4 (0.3)	2.2 (1.5)^†^^‡^
Boston naming test	12.9 (2)	11.6 (3)	9.6 (4)^†^
Lexical fluency (D-words)	15.8 (4)	15.8 (5)	10.1 (6)^†^
Semantic fluency (animals)	23.8 (6)	24.2 (6)	15.3 (7)^†^^‡^
Forward digit span	7.1 (1)	7.1 (1)	5.0 (2)
Backward digit span	5.9 (1)	5.1 (1)	3.3 (1)^†^^‡^
Stroop color naming	14.3 (1)	13.7 (1)	11.3 (4)
Stroop interference	91.4 (14)	81.8 (17)	63.4 (24)^†^
Modified trails time	22.4 (12)	26.4 (9)	55.3 (34)^†^^‡^
Modified rey figure copy	15.7 (0.7)	15.4 (1.3)	14.4 (1.3)^†^^‡^
**Volumetric MRI**			
Whole brain volume (cm^3^)	617 (68)	589 (75)	492 (81)^†^^‡^
Left frontal cortex (cm^3^)	54 (6)	50.7 (6)	40.1 (9)^†^^‡^
Right frontal cortex (cm^3^)	51.7 (6)	48.2 (5)	39.1 (10)^†^^‡^
Left temporal cortex (cm^3^)	29.0 (3)	27.3 (3)	22.7 (3)^†^
Right temporal cortex (cm^3^)	28.7 (3)	26.7 (3)	22.7 (5)^†^^‡^
Bifrontal average FA	0.45 (0.03)	0.46 (0.02)	0.37 (0.27)^†^^‡^
Left frontal average FA	0.43 (0.03)	0.44 (0.03)	0.35 (0.27)^†^^‡^
Right frontal average FA	0.42 (0.03)	0.43 (0.03)	0.35 (0.27)^†^^‡^
Corpus callosum genu FA	0.56 (0.3)	0.56 (0.2)	0.44 (0.09)^†^^‡^

† *Indicates values significantly differed from control (p < 0.05, uncorrected)*.

‡ *Indicates values significantly differed from asymptomatic GRN mutation carriers (p < 0.05, uncorrected)*.

*CCL18, chemokine C-C motif ligand 18; CDR-FTLD SB, frontotemporal lobar degeneration specific clinical dementia rating scale sum of boxes score; CGIS, Clinical Global Impression Severity score; CRP, C-reactive protein; CVLT, California Verbal Learning Test; D^1^, D prime; DTI, diffusion tensor imaging; FAQ, Functional Activities Questionnaire; IL-18, interleukin 18; LBP, lipopolysaccharide binding protein; MMSE, Mini-Mental Status Exam; sCD14, soluble Cluster of Differentiation 14; sCD163, soluble Cluster of Differentiation 163; SD, standard deviation*.

### Plasma Marker Levels

Six selected plasma biomarkers of innate immune activity were measured (sCD163, CCL18, sCD14, LBP, CRP, IL18) (Figure 1). No other fluid biomarkers were analyzed for this study. Our biomarkers of interest were not uniformly available in commercial multiplex panels, so each biomarker was measured individually by ELISA at a contract research organization (CRO) (Assaygate, Inc., Ijamsville, MD). Biomarkers were measured from frozen plasma, which was collected within 90 days of clinical and radiographic data. Samples were deidentified and randomly arranged across cohorts before being sent to the CRO.

### MRI Image Acquisition and Processing

The majority of T1 MR and diffusion tensor brain images were obtained at UCSF via the same 3 Tesla Siemens Tim Trio system with a 12-channel head coil. A single control participant's T1 images were obtained at UCSF via a 3 Tesla Siemens Prisma System under an equivalent harmonized protocol. Image acquisition was performed under previously published parameters (16, 17) as discussed in Supplemental Methods. After a quality control review, 22 controls, 14 asymptomatic carriers, and 11 symptomatic carriers had valid T1 weighted MR imaging within 90 days of their plasma collection, and of these cases 17 controls, 12 asymptomatic carriers, and 10 symptomatic carriers had valid diffusion tensor imaging.

T1-weighted images underwent segmented in SPM12 (Wellcome Trust Center for Neuroimaging, London, UK, www.fil.ion.ucl.ac.uk/spm) were used to create a study specific template using DARTEL (18), smoothed using a 6 mm Gaussian FWHM kernel. Each study participant's segmentation was inspected to ensure the robustness processing. Volumes in specific brain regions of interest (ROI) were calculated by transforming a standard parcellation atlas (19) into ICBM space and summing all gray matter within each parcellated region.

Diffusion images initially underwent denoising (20) and were realigned using the FSL MCFLIRT algorithm (21). The Dipy non-linear least-squares algorithm (22) was used to calculate diffusion tensors (DT), and a study specific template was created through iterative linear and non-linear registration of diffusion tensor images. Once in groupwise space, DT images were diagonalized into eigenvectors from which fractional anisotropy (FA) maps were calculated. Frontal and temporal regions of interest with extracted using the ICVM-DTI-81 white matter labels and tract atlas (23).

### Volumetric Analysis

Voxel-based statistics were performed in SPM12 and used to investigate relationships between brain volume and plasmas marker of interest. Contrasts modeled an interaction between *GRN* mutation carrier status and each inflammatory biomarker level in determining burden of atrophy across all cases. Clusters and voxels with a SPM familywise error (FWE) corrected *p*-value <0.05 were considered significant. To further explore the relationship between brain volume and plasma markers, each cluster with significant constituent peak voxels in VBM analysis was used to define a tailored (ROI). Composite frontotemporal regions of interest were also created by summing volumes within individual right frontal, left frontal, right temporal, and left temporal gray mater regions of interest from a standard parcellation atlas (19) transformed into ICBM space. Linear regression analysis was used in *GRN* mutation carriers to compare plasma markers with volume in the tailored ROIs, the whole brain (excluding CSF spaces), and in composite frontal and temporal ROIs. Linear regression analysis was also used in *GRN* mutation carriers to compare plasma biomarkers with gray matter asymmetry (the absolute value of total right gray matter volume subtracted from total left gray matter volume) and white matter asymmetry (the absolute value of total right white matter volume subtracted from total left white matter volume). Additionally, linear regression analysis was used to model an interaction between biomarkers and clinical status (symptomatic vs. asymptomatic) in determining volume in our tailored ROIs. Age, sex, and total intracranial volume (TIV) were used as covariates in all voxel-based statistics and linear regression analyses.

### Diffusion Tensor Imaging (DTI) Analyses

Using a hypothesis-based approach (based on the distribution of white matter findings in our VBM analysis) a composite bifrontal lobar region of interest (ROI) was generated, averaging fractional anisotropy (FA) values across the bilateral superior longitudinal fasciculus, the bilateral cingulum cingulate, and the genu of the corpus callosum. Linear regression models assessed the correlation between bifrontal FA and individual plasma markers. Additional analyses focused on LBP (the sole marker with a linear relation to bifrontal FA) and FA in constituent regions of interest: the right and left frontal ROIs (superior longitudinal fasciculus and cingulum bundle), the right and left superior longitudinal fasciculus, the right and left cingulum bundle, and the genu of the corpus callosum. Age and sex were used as covariates in all VBM and linear regression analyses. A familywise error correction (Šidák correction) was applied to analysis across multiple constituent ROIs.

### Clinical Assessments

Clinical disease severity was primarily assessed using the FTD-specific clinical dementia rating scale sum of boxes score (FTLD-CDR SB) (24). Clinical severity was also assessed vis the Functional Activities Questionnaire (FAQ) (25), the Clinical Global Impression Severity score (CGIS) (26), the Mini-Mental State Examination (MMSE) (27) the 9 item California Verbal Learning Test (CVLT) (28), Boston Naming Test (BNT) (29), phonemic fluency (D-words/minute) (30), semantic fluency (animals/minute) (31), digit span (forward and backward) (32), the Stroop color naming and inhibition tasks (33), the Modified Trails Test (30), and the Modified Rey Figure Copy (30). A familywise error correction (Šidák correction) was then applied (across multiple cognitive measures) as a follow up sensitivity analysis.

### Statistical Analysis

Statistical analysis was performed using Stata®, version 14.2. The normality of plasma marker distributions was assessed via the skewness kurtosis test (34). Non-normally distributed values (LBP, CRP, and IL18) were compared using non-parametric methods, including the Kruskal-Wallis test followed by *post-hoc* Wilcoxon pairwise rank sum testing. All other markers (sCD163, CCL18, and sCD14) were compared between our 3 groups with Analysis of Variance (ANOVA), followed by a *post-hoc* pairwise Tukey test to correct for multiple pairwise comparisons. We did not additionally adjust for multiple comparisons between differing biomarkers, as individual group-wise findings should be allowed to support each other rather than detract from each given the biological interrelation of biomarkers expressed in Figure 1. Additionally, our project was intended to identify potential biomarkers of interest and their interrelationships for future studies, so we elected to minimize the chance of type II error. Linear regression models were fit to examine the relationship between FTLD-CDR SB or other clinical assessments and individual plasma biomarkers. Analyses were restricted to symptomatic carriers for measures that were at floor in asymptomatic carriers (CDR-FTLD SB, CGIS). Age and sex were included as covariates in all models. As a follow up sensitively analysis, a familywise error correction (Šidák correction) was applied for regression analyses repeated across six different biomarkers.

### Patient and Public Involvement

This research study utilized clinical data and specimens previously collected at the University of California Memory and Aging Center. Patients were therefore not directly involved in determining the design, research questions, outcome measures, recruitment, conduct or assessment of burden in this study.

## Results

### Group Differences in Clinical, Biomarker, and Imaging Measures

**Figure 2:**
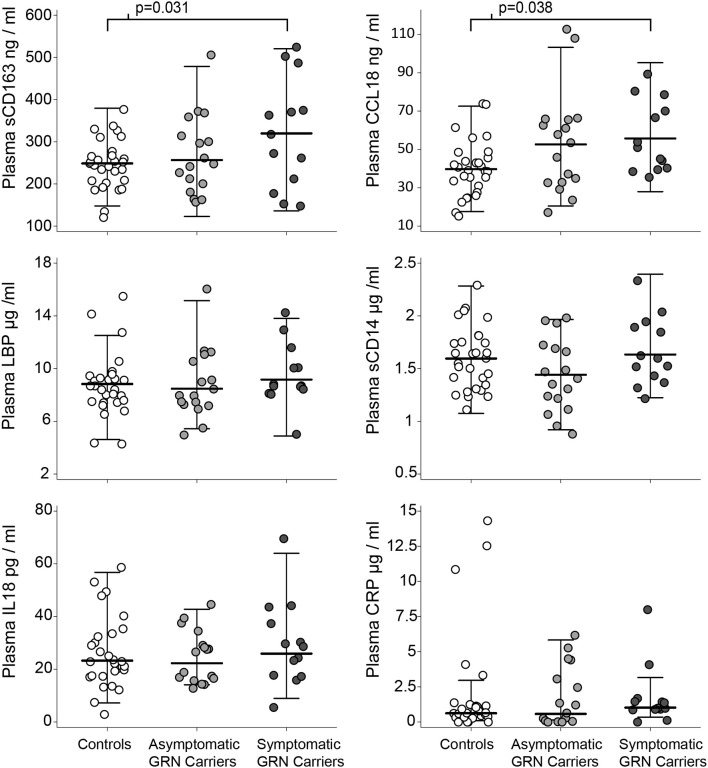
Plasma markers of innate immunity by clinical group. Circles represent individual people in the study. Whiskers represent the highest and lowest adjacent values (±1.5 × interquartile range) with a median line in between. The *p*-values associated with brackets for the sCD163 and CCL18 plots are *post-hoc* pairwise Tukey tests (after ANOVA *p* < 0.05) between controls and symptomatic carriers.

Patient demographics are summarized in Table 1. Normal controls, asymptomatic *GRN* mutation carriers, and symptomatic *GRN* carriers did not differ significantly in age. Asymptomatic mutation carriers had slightly higher CDR-FTLD-SB than controls (*p* = 0.0011 Tukey corrected), but these two cohorts did not differ in any other collected clinical or radiographic measures. Symptomatic FTD-*GRN* patients differed from controls and asymptomatic carriers across the majority of clinical and radiographic measures assessed (Table 1). Symptomatic *GRN* mutation carriers displayed high sCD163 (*p* = 0.031 Tukey corrected) and CCL18 (*p* = 0.038 Tukey corrected) concentrations relative to controls (Figure 2, Table 1). Plasma concentrations of CD14, LBP, CRP, or IL-18 did not differ between groups.

### Inflammatory Biomarkers Reflect Clinical Severity

**Figure 3:**
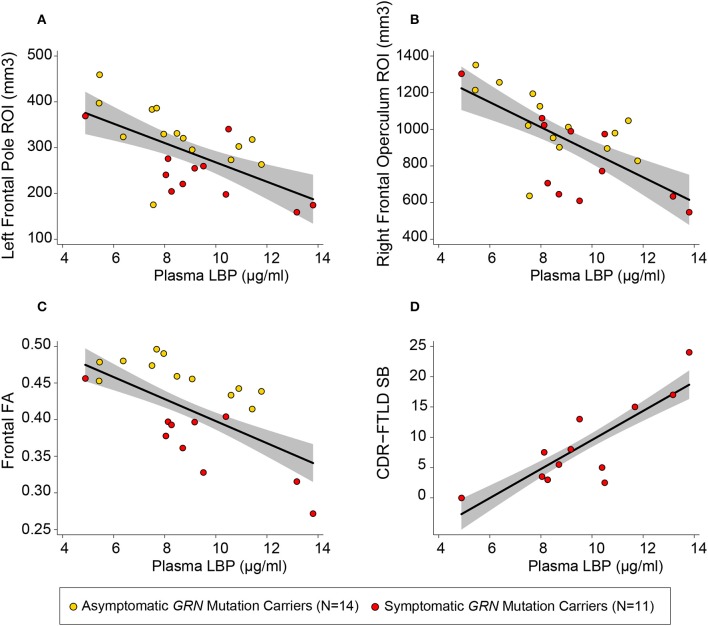
Plasma LBP correlates with white matter integrity and clinical severity. Circles represent uncorrected plotted values for individual GRN mutation carriers within our study, with yellow circles representing asymptomatic individuals and red circles representing symptomatic individuals. The left frontal **(A)** and right frontal **(B)** regions of interest (ROIs) depicted were defined by the left frontal pole white matter and right inferior frontal operculum white mater clusters described in Table 2. A linear regression line [controlling for age and sex, as well as total intracranial volume (TIV) for models with volumetric measures] with a gray 95% confidence interval has been superimposed on each plot [**(A)** *R*^2^ = 0.85, *p* = 0.024 uncorrected; **(B)** *R*^2^ = 0.86, *p* < 0.0005 uncorrected; **(C)** *R*^2^ = 0.59, *p* = 0.009; **(D)** *R*^2^ = 0.75, *p* = 0.003 uncorrected].

Among patients with symptomatic disease, there was a positive linear relationship between plasma LBP concentrations and CDR-FTLD SB (b = 2.27 CDR units/μg LBP/ml plasma, 95% CI [1.02, 3.51], *p* = 0.003 uncorrected, R^2^ = 0.76) (Figure 3) and the FAQ (b = 3.74 FAQ units/ μg LBP/ml Plasma, 95% CI [1.36, 6.11], *p* = 0.007 uncorrected, R^2^ = 0.68). There was a trend toward correlation between CRP levels and FTLD-CDR SB, but that finding did not survive multiple comparisons (R^2^ = 0.67, *p* = 0.01 uncorrected). There was no relationship between CDR-FTLD and other plasma markers including sCD163, sCD14, CCL18, and IL-18 in symptomatic *GRN* mutation carriers. Plasma biomarker levels did not correlate with other clinical assessments.

### Plasma Biomarkers and Brain Atrophy

**Figure 4:**
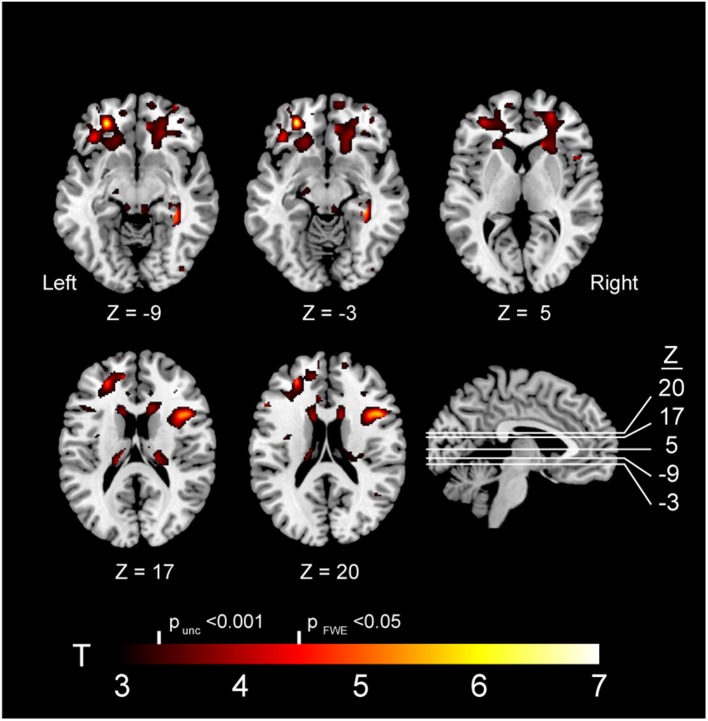
Brain atrophy associated with elevated plasma LBP in *GRN* carriers. Abbreviations: FWE, familywise error; LBP, lipopolysaccharide; unc, uncorrected. Note: The depicted clusters were obtained via voxel-based morphometry (VBM) analysis modeling an interaction between *GRN* mutation carriers status and plasma LBP in determining atrophy in our study cohort (including controls and mutation carriers).

VBM revealed multiple frontal-predominant clusters (in white matter more than gray matter) where elevated markers of inflammation were associated with more severe atrophy in *GRN* mutation carriers (Table 2). Elevated LBP was associated with decreased white matter in the left frontal pole (*p* < 0.001, FWE corrected), right inferior operculum (*p* = 0.006, FWE corrected), and right anterior internal capsule (*p* < 0.001, FWE corrected) (Figure 4). Elevated LBP was also associated with decreased gray matter in the left anterior frontal pole (*p* = 0.002, FWE corrected). There was a nearly significant association between elevated plasma sCD14 and decreased white matter integrity in the left middle temporal gyrus (*p* = 0.05, FWE corrected), and elevated plasma IL-18 was associated with decreased gray matter in the middle occipital gyrus (*p* = 0.033, FWE corrected).

**Table 2:** Brain atrophy is associated with elevated plasma markers in GRN carriers.

**Marker**	**Region**	**Description**	**Cluster**	**Peak voxel**	**Voxel location (mm)**	**X**	**Y**	**Z**
			***p* (FWE corrected)**	**T score**	**Z score**			
LBP	White matter	Left frontal pole	0.000	6.59	5.41	−27	45	−11
LBP	White matter	Right inferior operculum	0.006	5.72	4.87	45	15	20
LBP	White matter	Right anterior internal capsule and adjacent white matter	0.000	4.58	4.09	17	26	5
LBP	Gray matter	Left anterior inferior frontal gyrus and orbitofrontal gyrus	0.002	4.5	4.03	−33	30	−12
sCD14	White matter	Left middle temporal gyrus	0.050	5.18	4.52	−35	5	−41
IL-18	Gray matter	Left middle occipital gyrus	0.033	5.35	4.63	−36	−81	39

*FWE, familywise error; IL-18, interleukin 18; LBP, lipopolysaccharide binding protein; sCD14, soluble Cluster of Differentiation 14; VBM, voxel based morphometry. A FWE corrected alpha ≤ 0.05 was used to define significance*.

In order to better assess the relationship between plasma LBP and white matter integrity, two additional volumetric ROIs (left frontal pole white matter and right inferior operculum white matter) were generated from significant clusters in VBM analyses. Within *GRN* carriers, there was a linear relationship between elevated plasma LBP and more severe white matter atrophy in a left frontal pole ROI white matter ROI (b = −8.59 mm^3^/mg LBP/ ml plasma, 95% CI [−10.6, −1.26], R^2^ = 0.84, *p* = 0.024 uncorrected) and right inferior frontal operculum white matter ROI (b = −38.9 mm^3^/mg LBP/ ml plasma, 95% CI [−63.6, −14.2] R^2^ = 0.86, *p* < 0.001 uncorrected,), LBP, sCD14, and IL-18 did not correlate with whole brain volume or frontotemporal composites. All six plasma biomarkers of interested did not correlate with gray matter asymmetry or white matter asymmetry. There was no interaction between plasma LBP and clinical status (symptomatic vs. asymptomatic) in determining volume in our tailored volumetric ROIs in mutation carriers.

### Plasma Biomarkers and White Matter Integrity

We used DTI measurements of fractional anisotropy (FA) to further investigate the relationship between plasma LBP and white matter integrity. Elevated LBP was related to decreased fractional anisotropy (FA) in the bilateral frontal lobes (b = −0.016 FA/μg/ml LBP, R^2^ = 0.59, *p* = 0.009 uncorrected) in *GRN* mutation carriers (Figure 3C, Table 3). LBP elevation was also associated with decreased FA within multiple constituent regions of interest, including a left frontal lobe composite region of interest (*p* = 0.004 uncorrected), a right frontal composite region of interest (*p* = 0.026 uncorrected), the genu of the corpus callosum (*p* = 0.027 uncorrected), the left cingulum cingulate (*p* = 0.003 uncorrected), the right cingulum cingulate (*p* = 0.02 uncorrected), and the left superior longitudinal fasciculus (*p* = 0.015 uncorrected). The association between LBP and FA remained significant in the left cingulum cingulate and left frontal lobe composite ROI after correction for multiple comparisons.

**Table 3:** Bifrontal DTI measures correlate with plasma LBP in GRN mutation carriers.

	**b (FA /μg/ml)**	**[95% conf.]**	**R^**2**^**	***p***
Bi-frontal (FA)	−0.013	(−0.023, −0.004)	0.59	0.009
**Constituent regions of interest**				
Left frontal (FA)	−0.013	(−0.022, −0.005)	0.61	0.004^†^
Right frontal (FA)	−0.01	(−0.019, −0.001)	0.57	0.026
Genu of the corpus callosum	−0.019	(−0.035, −0.003)	0.47	0.027
Left cingulum cingulate	−0.018	(−0.028, −0.007)	0.61	0.003^†^
Right cingulum cingulate	−0.011	(−0.020, −0.002)	0.61	0.02
Left superior longitudinal fasciculus	−0.009	(−0.016, −0.002)	0.54	0.015
Right superior longitudinal fasciculus	−0.009	(−0.0179, 0.0002)	0.46	0.056

† *Denotes findings that remained significant after a follow up familywise error correction; alpha ≤ 0.0073*.

*DTI, diffusion tensor imaging; CI, confidence interval; FA, fractional anisotropy; LBP, lipopolysaccharide binding protein*.

*The presented regression analyses results were controlled for age and sex, and the presented R^2^ values apply to the entire model. All p-values are presented in their uncorrected form*.

## Discussion

We identified a direct correlation between disease severity and a plasma mediator of the peripheral innate immune response (LBP) in *GRN* mutation carriers (symptomatic and asymptomatic). Additionally, we identified an elevation in plasma biomarkers of ongoing monocyte turnover (sCD163) and chronic monocyte activation (CCL18) in symptomatic *GRN* mutation carriers (though not asymptomatic mutation carriers) relative to controls.

Our results potentially represent early evidence for a neuro-inflammatory model of FTD-GRN progression, in which the peripheral innate immune systems directly contributes to brain pathology. In particular, our findings indicate a possible relationship between the peripheral immune system and white matter disease in *GRN* mutation carriers. Alternatively, it is possible that our results represent parallel autonomous processes in the peripheral immune system and CNS or peripheral inflammation in response to CNS neuropathology.

White matter T2 hyperintensities are well described in FTD-GRN, and neuroinflammation has previously been proposed as a mechanism for these lesions (7). In Grn^−/−^ mice, microglia exert a pathogenic effect on neurons (8) due in part to excessive compliment-mediated synaptic destruction (35). A similar process of synaptic destruction, either through direct infiltration of monocytes or secondary activation of glial cells (36), may explain the relationship between peripheral inflammation and white matter in our study. Activation of the peripheral immune system, particularly of monocytes, is known to induce secondary activation of microglia (36) outside of FTD models. Additionally, FTD-*GRN* may involve blood-brain barrier dysfunction (5), which could increase the interaction between peripheral and central innate immune cells. White matter change in FTD-*GRN* may also reflect the anatomical distribution of perivascular macrophages (on the front line of interaction with the peripheral immune system) aligning deep penetrating small vessels.

Plasma LBP is an acute phase reactant released by the liver (8) and is unlikely to faithfully track monocyte activation downstream. The correlation between plasma LBP and white matter changes may, however, reflect a specific upstream mechanism of monocyte activation in *GRN* mutation carriers. Plasma LBP is an essential factor for activation of monocytes by bacterial endotoxins via the toll-like receptor 4 (TLR4) (8). The correlation between LBP and disease may therefore reflect the role of external antigens, such as endotoxins from gut microbes, in promoting FTD-*GRN*. *Grn* knockout (*Grn*^−/−^) mice exhibit poor clearance of bacterial pathogens and hyper-activation to bacterial LPS via TLR4 (3). Patients with GRN haploinsufficiency may similarly experience dysfunctional inflammation, with poor clearance of bacterial pathogens despite excessively robust monocyte activation. Under this model, GRN mutation carriers and healthy controls would experience very different sustained monocyte responses in reaction to the same inflammatory challenge. This may explain why LBP correlated with disease in mutation carriers, even though LBP levels were similar to controls.

Plasma sCD163 and CCL18, unlike LBP, are directly released from monocytes and represent differing aspects of monocyte function. After monocytes are activated (by LPS or other triggers), they cleave membrane-bound CD163 and release the soluble form (sCD163) (9). In models of encephalitis, sCD163 has been used as a proxy for monocyte migration to the site of brain inflammation (37). After stimulation, classical monocytes either leave circulation or transition to intermediate and eventually to non-classical forms (11). A sustained peripheral shift in monocyte phenotypes, away from classical monocyte forms, is a hallmark of a variety of chronic infections and autoimmune diseases (38). CCL18 is released chiefly by myeloid cells and has long been described as a marker of non-classical monocytes (12). Taken together, elevation of sCD163 and CCL18 in symptomatic FTD-GRN hint at a chronic inflammatory state, in which classical peripheral monocytes are chronically migrating to sites of inflammation and transitioning to non-classical forms in parallel. In the context of our findings pertaining to LBP, this chronic activation state may be driven in part by bacterial endotoxin.

This study had several limitations. Voxel-based morphometry is a technique that is chiefly used to analyze gray matter volumes, and the majority of our volumetric findings are in white matter regions of interest. Fortunately, fractional anisotropy provided an alternate modality that confirmed associations between LBP and white matter integrity. Additionally, heterogeneous asymmetry may have limited our ability to find associations between brain volume and biomarkers using a voxel-based approach. For this reason, it is possible that larger follow up studies would identify more extensive bifrontal volume regions associated with plasma marker of innate immune activation. Our findings were limited to a relatively small cohort of mutation carriers, given the rarity of *GRN* haploinsufficiency and the accessibility of biospecimens. Interpretation of our findings would greatly benefit from replication in a separate larger cohort. Our imaging analysis merged all mutation carriers (pre and post symptomatic) into one cohort to make inferences about the entire clinical spectrum of *GRN* haploinsufficiency. A larger clinical study will allow for greater distinction between immune phenomena in asymptomatic mutation carriers and FTD-*GRN*. Because our data were from single time points in each participant, it is difficult to discern what proportion of variance in each plasma marker is due to sustained inflammation vs. short-term fluctuations in inflammatory markers related to transient stressors. Longitudinal data would therefore improve our ability to make inferences about the relationship between inflammatory markers and disease severity in *GRN* mutation carriers. Additionally, our study used a small set of relatively specific plasma markers to make specific inferences about differing aspects of myeloid cell behavior and activity. Follow-up studies would ideally include methods that more directly assess innate immune cells in *GRN* mutation carriers. Future studies should seek to assess less specific biomarkers of inflammation, given previous evidence of high peripheral tumor necrosis factor alpha (TNF-α) (4) and Interleukin 6 (IL-6) (39) in patients with *GRN* haploinsufficiency.

This study highlights the potential value of blood biomarkers of innate immunity as tools to understand disease pathogenesis in *GRN* mutation carriers. Analysis of longitudinal datasets such as Longitudinal Evaluation of Familial Frontotemporal Dementia Subjects (LEFFTDS) and the Genetic FTD Initiative (GENFI) will ultimately help to determine the temporal relationship between inflammatory biomarker elevation, clinical severity, and neuroimaging changes in *GRN* mutation carriers. The current findings also suggest that clinical trials of agents that reduce monocyte/macrophage or microglial activation might help to elucidate the relationship between inflammation and disease in *GRN* mutation carriers and could potentially be used to prevent or treat FTD-*GRN*.

## Data Availability

The datasets generated for this study are available on request to the corresponding author.

## Ethics Statement

This study was approved by the UCSF Institutional Review Board (IRB) and all participants gave written informed consent in accordance with the Declaration of Helsinki.

## Author Contributions

PL, LM, HR, and AB: design and conceptualization of the study, analysis and interpretation of the data, and drafting and revising the manuscript for intellectual content. ZM, IA, AS, HH, JR, and YC: analysis and interpretation of the data and drafting and revising the manuscript for intellectual content. PM, BM, and JK: analysis and interpretation of the data. JZ and MM: design and conceptualization of the study and analysis and interpretation of the data. AK and RP: design and conceptualization of the study. Statistical analysis was conducted by PL, PM, and IA.

### Conflict of Interest Statement

LM received compensation for serving on the advisory board of Tiake Therapeutics. MM owns stock in Neuraltus Pharmaceuticals. AB receives research support from NIH U54NS092089, R01AG031278, R01AG038791, R01AG032306, R01AG022983, The Tau Research Consortium, The Bluefield Project to Cure Frontotemporal Dementia, Corticobasal Degeneration Solutions, and the Alzheimer's Association. He has served as a consultant for Abbvie, Celgene, Ionis, Janssen, Merck and Novartis, and received research support from Avid, Biogen, BMS, C2N, Cortice, Forum, Genentech, Janssen, Pfizer, Eli Lilly, Roche, and TauRx, He holds Stock Options in Alector and Delos. The remaining authors declare that the research was conducted in the absence of any commercial or financial relationships that could be construed as a potential conflict of interest.
