# Supplementary material for: Peripheral Innate Immune Activation Correlates With Disease Severity in GRN Haploinsufficiency
Source: Front Neurol. 2019 Sep 18;10:1004. doi: 10.3389/fneur.2019.01004 (PMC6759464; doi:10.3389/fneur.2019.01004)
Supplement: Supplementary file 1 [file Table_1.DOCX]

**Supplemental Methods:**

**Image acquisition**

The majority of volumetric images were acquired on a 3.0 T Siemens Tim Trio system (Siemens, Iselin, NJ) , though a single control participant’s images were acquired with a 3.0T Siemens Prisma System. Volumetric MPRAGE sequences at UCSF was used to acquire T1-weighted images of the entire brain (Sagital slice orientation; slice thickness = 1.0 mm; slices per slab = 160; in-plane resolution = 1.0x1.0 mm; matrix = 240X256; TR = 2,300 ms; TE = 2.98 ms for Trio and 2.9 for Prisma; TI = 900 ms; flip angle = 9°).

Diffusion sequences were acquired using the following previously published parameters(1):

"MR images were acquired on a 3 Tesla Siemens Tim Trio system equipped with a 12-channel head [coil](https://www.sciencedirect.com/topics/neuroscience/coil) at the UCSF Neuroscience Imaging Center. Diffusion sequences were acquired using the following parameters: TR/TE 8200/86 ms; B  =  0 image and 64 directions at B  =  2000 s/mm^2^; FOV 220 × 220 mm^2^ and 2.2 mm thick slices; [matrix](https://www.sciencedirect.com/topics/medicine-and-dentistry/matrix-biology) 100 × 100 with 60 slices yielding 2.2 mm^3^ isotropic voxels/(TR/TE 8000/109 ms; B  =  0 image and 64 directions at B  =  2000 s/mm^2^; FOV 220 × 220 mm^2^ and 2.2 mm thick slices; matrix 100 × 100 with 55 slices yielding 2.2 mm^3^ isotropic voxels).”

**Volumetric image processing and template creation**

All T1-weighted images were visually inspected prior to use, and images with excessive motion or image artifact were excluded from this study. Tissue was segmented using unified segmentation(2) with SPM12 (Wellcome Trust Center for Neuroimaging, London, UK, [www.fil.ion.ucl.ac.uk/spm](http://www.fil.ion.ucl.ac.uk/spm)). We created a study specific template by warping each study participant’s T1-weighted image template using *Diffeomorphic Anatomical Registration using Exponentiated Lie algebra* (DARTEL)(3). We then modulated and smoothed grey and white matter tissues within our template using a Gaussian kernel with 6~mm full width at half maximum (FWHM). Each study participant’s segmentation was inspected to ensure the robustness processing. Volumes in specific brain regions of interest (ROI) were calculated by transforming a standard parcellation atlas (4) into ICBM space and summing all gray matter within each parcellated region.

**Diffusion tensor image processing and template creation**

Diffusion images initially underwent denoising(5) and were then realigned using the FSL MCFLIRT algorithm(6). The non-linear least-squares algorithm was used in Dipy (7) to calculate diffusion tensors. A study specific template was created through iterative linear and non-linear registration of diffusion tensor images. Once in group-wise space, diffusion tensor images were diagonalized into eigenvectors from which fractional anisotropy maps were calculated. Frontal and temporal regions of interest with extracted using the ICVM-DTI-81 white matter labels and tract atlas (8)

**References:**

1. Elahi FM, Marx G, Cobigo Y, Staffaroni AM, Kornak J, Tosun D, et al. Longitudinal white matter change in frontotemporal dementia subtypes and sporadic late onset Alzheimer’s disease. NeuroImage Clin. 2017 Jan 1;16:595–603.

2. Ashburner J, Friston KJ. Unified segmentation. Neuroimage. 2005 Jul 1;26(3):839–51.

3. Ashburner J. A fast diffeomorphic image registration algorithm. Neuroimage. 2007 Oct 15;38(1):95–113.

4. Desikan RS, Ségonne F, Fischl B, Quinn BT, Dickerson BC, Blacker D, et al. An automated labeling system for subdividing the human cerebral cortex on MRI scans into gyral based regions of interest. Neuroimage. 2006 Jul;31(3):968–80.

5. Veraart J, Fieremans E, Novikov DS. Diffusion MRI noise mapping using random matrix theory. Magn Reson Med. 2016 Nov;76(5):1582–93.

6. Jenkinson M, Beckmann CF, Behrens TEJ, Woolrich MW, Smith SM. FSL. Neuroimage. 2012 Aug;62(2):782–90.

7. Garyfallidis E, Brett M, Amirbekian B, Rokem A, van der Walt S, Descoteaux M, et al. Dipy, a library for the analysis of diffusion MRI data. Front Neuroinform. 2014 Feb;8:8.

8. Mori S (Susumu), Crain BJ. MRI atlas of human white matter. Elsevier; 2005. 239 p.
